# Supplementary material for: Naïve plant communities and individuals may initially suffer in the face of reintroduced megafauna: An experimental exploration of rewilding from an African savanna rangeland
Source: PLoS One. 2021 Apr 6;16(4):e0248855. doi: 10.1371/journal.pone.0248855 (PMC8023473; doi:10.1371/journal.pone.0248855)
Supplement: Attachment — Submitted filename: young et al.docx [file pone.0248855.s001.docx]

Review

Title: Naïve plant communities and individuals may initially suffer in the face of reintroduced

megafauna: an experimental exploration of rewilding from an African savanna

rangeland

Authors: Truman P. Young, Harry Wells, Duncan Kimuyu, Wilfred Odadi, Amelia Wolf

Overview:

Young et al. present data that emerged from a long-term herbivory exclosure experiment. They show that when plants are protected from the grazing activities of mega herbivores, they lose their defenses, become naïve and more vulnerable to the subsequent return of grazers. This paper is brief, clear and the data supports their conclusions well. The results they find here are important to a large body of fundamental literature that studies the effects of herbivory on plant communities as well as the literature addressing the science of conservation and rewilding. I appreciate their attention to both.

The conclusions of this paper would benefit from an acknowledgment of the variability of time. Diversity of the plant communities is higher in exclosures after 25 years. I am not sure that would be the case if herbivores were excluded in perpetuity. I also feel that future work that includes genetic analysis, particularly on the acacia would be incredibly interesting especially given they can age the trees. Broadly, the paper is very well written. I have a few minor hesitations that will be described below in comments.

Comments:

1. Please always include line numbers to assist your reviewers.
2. I strongly encourage the authors to remove the value laden statements. This paper is submitted as a ‘research paper’ not as an opinion piece or synthesis in which the reader may expect presentation of the authors opinions. As such, I also encourage them to remove the quote at the end of the discussion. The data and conclusions of this work are clear, compelling and speak for themselves. Given this is a research article, please allow your readers to make their own interpretation regarding the value of rewilding and its implications for conservation.

For example, in the abstract: “In summary, the native communities that we observe in defaunated landscapes may be very different from their pre-defaunation states, and we are likely to see some large (and potentially troubling) changes to these plant communities upon rewilding with large herbivores.” Potentially troubling to whom? This sentence otherwise clearly describes your results, but its strength is lost when it becomes a statement of your opinion.

3. Table 1 is more of a list than a comparative table. I suggest moving that to an appendix if possible.
4. Table 2 appears unformatted. It is also not necessary given the statistics presented in the figures. I encourage you to remove it or format it formally.
5. Can you please remind the readers what O, C, MW and MWC designate? Do this for Figure 3 and Table 3.
6. Figure quality appears low. I assume the journal will require higher resolution images. They were tough to read (esp fig 3).
7. Replication should be stated clearly in the methods and presented again in each figure caption. Is it three given six treatments and 18 plots? This is not clear.

reviewed by Jennifer Adams Krumins
