# Supplementary material for: Naïve plant communities and individuals may initially suffer in the face of reintroduced megafauna: An experimental exploration of rewilding from an African savanna rangeland
Source: PLoS One. 2021 Apr 6;16(4):e0248855. doi: 10.1371/journal.pone.0248855 (PMC8023473; doi:10.1371/journal.pone.0248855)
Supplement: Attachment — Submitted filename: Responses to reviewers PONE-D-20-34917.docx [file pone.0248855.s002.docx]

Dear Editors,

Thank you for inviting a revision of ms PONE-D-20-34917, “Naïve plant communities and individuals may initially suffer in the face of reintroduced megafauna: an experimental exploration of rewilding from an African savanna rangeland”. Below are our responses (in Roman) to the comments of the reviewers and editors (in italics). Attached are both marked-up and clean versions of the revised ms (for some reason, the lines numbers on the marked-up version are misbehaving).

1.) *Please ensure that your manuscript meets PLOS ONE's style requirements, including those for file naming.*

Done (I think)

*2.) Please remove any funding-related text from the manuscript. Please include your amended statements within your cover letter; we will change the online submission form on your behalf.*

We have removed funding information from our Acknowledgements, and provide here a new version of our Funding Statement:

“The KLEE exclosure plots were built and maintained by grants from the James Smithson Fund of the Smithsonian Institution (to A.P. Smith), The National Geographic Society (Grants 4691-91, 9106-12, and 9986-16), The National Science Foundation (LTREB DEB 97-07477, 03-16402, 08-16453, 12-56004, 12-56034, and 19-31224) and the African Elephant Program of the U.S. Fish and Wildlife Service (98210-0-G563) (to T.P. Young, C. Riginos, and K.E. Veblen). The funders had no role in study design, data collection and analysis, decision to publish, or preparation of the manuscript.”

*3.) We note that you have stated that you will provide repository information for your data at acceptance. Should your manuscript be accepted for publication, we will hold it until you provide the relevant accession numbers or DOIs necessary to access your data. If you wish to make changes to your Data Availability statement, please describe these changes in your cover letter and we will update your Data Availability statement to reflect the information you provide.*

Please include this Data Availability statement”

“Data available from figshare: <https://doi.org/10.6084/m9.figshare.3175561.v1>”

*Review Comments to the Author*

*Reviewer #1: I appreciate having the opportunity to review this work, which is based on an account of the consequences of a serendipitous breach of the fence around an exclosure experiment in Kenya. After 20 years of protection from elephant browsing, the larger Acacia drepanolobium trees had apparently relaxed their defenses, with the defensive ant community having shifted to a less aggressive composition.*

This ms is not mainly about the fence break. the fence break is just one of three lines of evidence to support our thesis, and a secondary one at that, which we make clear in our Abstract and in the body of the ms

*There might also have been changes in spine length and leaf chemistry, though those were not reported.*

We more clearly cite published evidence from these same experimental plots for both of these effects, at Lines 230-231.

*Anyhow, the larger A. drepanolobium trees (>4 m high) had become “naïve” to elephants and seemingly also more palatable, and were damaged much more during the period that elephants had access to them than “experienced” trees that had been continually exposed to elephants. The authors use this case study to illustrate a thoughtful discussion about how rewilding a landscape through introductions of large herbivores might lead to an episode in which certain plant species are impacted much more than expected, because they have become “naïve”. This is a good point, and a helpful contribution to the current discussion on rewilding. I have two overall comments and several specific ones.*

Thank you!

*1). The scale dependence of megaherbivore effects need to be considered. This study is at the scale of 4 ha exclosure plots in a topographically homogenous landscape where the woody vegetation is strongly dominated by one species. At the spatial scale of an elephant home range (100s of square km), in a landscape with more diversity in topography (hills, rivers, plains) and plant community types, the services performed by elephants (nutrient cycling, seed dispersal, differential disturbance, tree felling that provides protection to seedlings beneath the recumbent canopy, etc., etc.) would be more evident. Certainly, as in this case, prolonged protection from large herbivores can be associated with reduced inducible defenses in plants, which are then vulnerable when large herbivores suddenly reappear, but some plant species are continuously vulnerable to large herbivores and persist in a source-sink dynamic system in which refugia are crucial. For example, baobab tree populations can be eradicated by elephants unless there are rocky hills on which some individuals persist and provide propagules that find their way into the areas below (Edkins et al. 2007). Also, riparian tree communities maintain diversity in the face of chronic elephant browsing by virtue of small stands persisting above steep river banks (du Toit et al. 2014). So, if rewilding projects involving large herbivores consider the issue of scale, and if topographic diversity is adequately incorporated into the reserve design, then the problem of plant naiveté should be greatly mitigated.*

Good point, and one that we have incorporated at Lines 291-295

*2). The discussion about the effects of cattle on herbaceous vegetation does not fit with the story about elephants selectively damaging tall A. drepanolobium trees. It is a distraction, especially for readers who do not know the functional traits of the various herbaceous species listed (Table 3, Fig. 3). I suggest keeping the focus on the woody vegetation.*

Again, the section on elephant damage is just one part of our compiled evidence (see also our comment above), and we strongly believe that the additional data from KLEE plots on differences in both woody and herbaceous plant communities are important aspects of the overall story.

*Page 3: In the introduction it is stated that rewilding is often associated with the elimination of non-native livestock, but I disagree. To the contrary, livestock species are commonly promoted as surrogates for extinct or rare wild species and are key players in well-known rewilding projects in Europe, for example, including Oostvaardersplassen in the Netherlands.*

This is a good point, and we have amended the ms (at Lines 35-36 and 63-64) to reflect this.

*Page 3: Spelling of Beschta.*

Corrected.

*Page 4: When elephants broke into the plots, for how long were they feeding there before the exclosure fence was repaired?*

Fixed at Lines 154-5.

*Table 3: This is difficult to interpret and seems tangential to the study.*

We have simplified Table 3, which report on of the three main results of this study.

*Figure 1: Are megaherbivores not wildlife? Where is the bar for the plots in which cattle were allowed to feed?*

We have reiterated our nomenclature of simplifying ‘meso-wildlife” to simply “wildlife” throughout this ms.

*Figure 3: This is difficult to interpret and seems tangential to the study.*

We have dropped this figure.

*Reviewer #2:*

*Young et al. present data that emerged from a long-term herbivory exclosure experiment. They show that when plants are protected from the grazing activities of mega herbivores, they lose their defenses, become naïve and more vulnerable to the subsequent return of grazers. This paper is brief, clear and the data supports their conclusions well. The results they find here are important to a large body of fundamental literature that studies the effects of herbivory on plant communities as well as the literature addressing the science of conservation and rewilding. I appreciate their attention to both.*

Thank you!

*The conclusions of this paper would benefit from an acknowledgment of the variability of time. Diversity of the plant communities is higher in exclosures after 25 years. I am not sure that would be the case if herbivores were excluded in perpetuity. I also feel that future work that includes genetic analysis, particularly on the acacia would be incredibly interesting especially given they can age the trees. Broadly, the paper is very well written. I have a few minor hesitations that will be described below in comments.*

We have added a sentence about our time scale, at Lines 279-280

*1. Please always include line numbers to assist your reviewers.*

Done.

*2. I strongly encourage the authors to remove the value laden statements. This paper is submitted as a ‘research paper’ not as an opinion piece or synthesis in which the reader may expect presentation of the authors opinions. As such, I also encourage them to remove the quote at the end of the discussion. The data and conclusions of this work are clear, compelling and speak for themselves. Given this is a research article, please allow your readers to make their own interpretation regarding the value of rewilding and its implications for conservation.*

We believe that the Discussion sections of mss are appropriate places to suggest implications of our findings, but we have removed the quote. (For now: we still like it, if the Editors do not mind.)

*For example, in the abstract: “In summary, the native communities that we observe in defaunated landscapes may be very different from their pre-defaunation states, and we are likely to see some large (and potentially troubling) changes to these plant communities upon rewilding with large herbivores.” Potentially troubling to whom? This sentence otherwise clearly describes your results, but its strength is lost when it becomes a statement of your opinion.*

We have amended this sentence.

*3. Table 1 is more of a list than a comparative table. I suggest moving that to an appendix if possible.*

Tables 1 and 3 (now Table 2) are indeed lists, and two of the central results of this study

*4. Table 2 appears unformatted. It is also not necessary given the statistics presented in the figures. I encourage you to remove it or format it formally.*

We dropped Table 2

*5. Can you please remind the readers what O, C, MW and MWC designate? Do this for Figure 3 and Table 3.*

We refer the reader to the Methods, but can repeat this information in each caption if the editors wish.

*6. Figure quality appears low. I assume the journal will require higher resolution images. They were tough to read (esp fig 3).*

We have dropped Figure 3.

*7. Replication should be stated clearly in the methods and presented again in each figure caption. Is it three given six treatments and 18 plots? This is not clear.*

Yes, it is three, and we have made this more clear in the Methods, and in the captions.

*While revising your submission, please upload your figure files to the Preflight Analysis and Conversion Engine (PACE) digital diagnostic tool, https://pacev2.apexcovantage.com/. PACE helps ensure that figures meet PLOS requirements. To use PACE, you must first register as a user. Registration is free. Then, login and navigate to the UPLOAD tab, where you will find detailed instructions on how to use the tool. If you encounter any issues or have any questions when using PACE, please email PLOS at figures@plos.org. Please note that Supporting Information files do not need this step.*
