# Supplementary material for: Naïve plant communities and individuals may initially suffer in the face of reintroduced megafauna: An experimental exploration of rewilding from an African savanna rangeland
Source: PLoS One. 2021 Apr 6;16(4):e0248855. doi: 10.1371/journal.pone.0248855 (PMC8023473; doi:10.1371/journal.pone.0248855)
Supplement: Attachment — Submitted filename: young et al R1.docx [file pone.0248855.s003.docx]

Review of R1

Title: Naïve plant communities and individuals may initially suffer in the face of reintroduced

megafauna: an experimental exploration of rewilding from an African savanna

rangeland

Authors: Truman P. Young, Harry Wells, Duncan Kimuyu, Wilfred Odadi, Amelia Wolf

Young et al. have satisfactorily addressed my comments and those of reviewer 1. I am happy to suggest this manuscript be accepted with minor caveats. I still do not understand why the lists are presented in the manuscript itself, and not as a formal table. I still hold they would be far more accessible if presented as a clean table and likely as a supplement. I also encourage the authors to double check the order and quality of their figures. I might be confused, but as they are presented in the revised manuscript, they appear to be ordered figure 2 before figure 1, and quality is still low.

reviewed by Jennifer Adams Krumins
