# Supplementary material for: Naïve plant communities and individuals may initially suffer in the face of reintroduced megafauna: An experimental exploration of rewilding from an African savanna rangeland
Source: PLoS One. 2021 Apr 6;16(4):e0248855. doi: 10.1371/journal.pone.0248855 (PMC8023473; doi:10.1371/journal.pone.0248855)
Supplement: Attachment — Submitted filename: R2R.docx [file pone.0248855.s004.docx]

5 Mar 2021

Dear Editors, (PLOS ONE)

Thank you for inviting a revision of ms PONE-D-20-34917, “Naïve plant communities and individuals may initially suffer in the face of reintroduced megafauna: an experimental exploration of rewilding from an African savanna rangeland”. Below are our responses (in Roman) to the comments of the reviewers and editors (in italics). I have also made some additional minor edits. Attached are both marked-up and clean versions of the revised ms.

***Reviewer #1:*** *My main comments from the first review have mostly been adequately addressed and, where not, it is a matter of opinion that the authors are entitled to maintain and the readers to evaluate. I have just a few minor comments:*

*The first two sentences of the last paragraph of the discussion seem odd. First, the question “Does rewilding need to minimize/eliminate the human footprint?” is moot since du Toit & Pettorelli (2019; referenced) outlined how rewilding is generally inclusive of people with nature (Table 1 of that paper).*

Yes, this has been said before, but is perhaps not yet widely accepted (especially in North America, as evidence by the following sentences). We have amended wording here and included specific reference to the du Toit & Pettorelli 2019).

*Second, the sentence “Africa as a test case …” is perhaps intended to read “Africa IS a test case”? Anyhow, extrapolating from the KLEE site to the whole of Africa is quite a stretch and so I suggest some rewording.*

Corrected and reworded
 *Line 375, correct to Ripple & Beschta (not “Breschta”).*

Done
 *Line 437 in the references please correct to du Toit JT (not “JY”).*

Done

***Reviewer #2:*** *Young et al. have satisfactorily addressed my comments and those of reviewer 1. I am happy to suggest this manuscript be accepted with minor caveats.*

*I still do not understand why the lists are presented in the manuscript itself, and not as a formal table. I still hold they would be far more accessible if presented as a clean table and likely as a supplement.*

My understanding is that the PLOS One production team will re-set Tables in their own table format in any case. I have replaced the text here with Excel Tables, if that helps. If PLOS One wants these is some other format, I am happy to do so. Please advise.

*I also encourage the authors to double check the order and quality of their figures. I might be confused, but as they are presented in the revised manuscript, they appear to be ordered figure 2 before figure 1, and quality is still low.*

This was due to a remnant reference (at Line 125) to the Figure 1 of the original submission, which was dropped on request of the reviewer. We have corrected this.

Figure quality re-checked. Production people will advise, I assume, if I am wrong.
